# Ferritin‐Based Nanomotors for Deep Glioblastoma Penetration and Enhanced Drug Delivery

**DOI:** 10.1002/mabi.70267

**Published:** 2026-09-30

**Authors:** Jun Ma, Ruochen Liu, Ibrahim Chamseddine, Jingjing Qiu, Shiren Wang

**Affiliations:** ^1^ Department of Biomedical Engineering Texas A&M University College Station Texas United States; ^2^ Department of Materials Science and Engineering Texas A&M University College Station Texas United States; ^3^ Department of Radiation Oncology Mass General Brigham Cancer Institute and Harvard Medical School Boston Massachusetts United States; ^4^ Department of Mechanical Engineering Texas A&M University College Station Texas United States; ^5^ Department of Industrial Systems and Engineering Texas A&M University College Station Texas United States

**Keywords:** cancer, drug‐delivery, nanomotor, tumor penetration

## Abstract

Poor penetration into tumor tissue remains a major barrier to effective nanomedicine delivery, particularly in glioblastoma multiforme (GBM). Here, nanoparticle transport was computationally studied to rationally design an ultrasmall chemotactic nanomotor that improves penetration into dense tumor tissue. The nanomotor comprises a targeting human heavy‐chain ferritin (HFn) nanocage and a catalytic cerium oxide (CeO_2_) component. Leveraging transferrin receptor‑1 overexpression on brain microvascular endothelial cells and GBM cells, HFn enables transport across an in vitro blood–brain barrier (BBB) model and accumulation in GBM spheroids. The CeO_2_ domain catalyzes hydrogen peroxide decomposition in the tumor‑mimicking microenvironment, generating oxygen and imparting directional propulsion along H_2_O_2_ gradients. In chemotaxis assays, the nanomotors actively migrated toward localized H_2_O_2_ sources and GBM cells embedded in Matrigel. In large U87 spheroids (>400 µm), HFn@CeO_2_ nanomotors penetrated deeply and distributed throughout the spheroid core, whereas non‑propelled HFn nanocarriers remained confined mainly to the periphery. Doxorubicin‑loaded nanomotors (HFn@CeO_2–_DOX) achieved substantially enhanced intratumoral DOX distribution and reduced IC_50_ (0.33 µM) compared with HFn–DOX (1.13 µM) and free DOX (1.75 µM) in GBM spheroids. These results provide a promising basis for future in vivo evaluation in brain tumor therapy.

## Graphical abstract

We report an oxygen‐propelled nanomotor composed of human heavy‐chain ferritin nanocage (HFn) and catalytic CeO_2_ nanoparticles to enhance tumor penetration and therapeutic efficacy. HFn efficiently traversed the blood‐brain barrier (BBB), while oxygen generated by CeO_2_‐catalyzed H_2_O_2_ decomposition propelled the nanomotor deep into tumor spheroids, improving intratumoral distribution and antitumor efficacy.

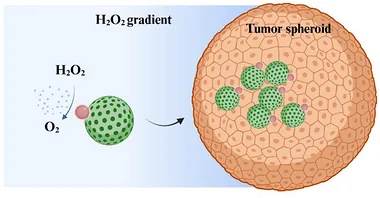

## 1 Introduction

As the most malignant brain tumor, glioblastoma multiforme (GBM) has an extremely poor 5‐year survival rate (6.8%) [1] and a median survival time of <16 months due to therapeutic resistance and a high recurrence rate [2, 3]. Clinical systemic chemotherapy shows only a small survival benefit (a 2‐month increase in median survival time) [2, 4]. In addition to cellular and genetic therapeutic resistance, the therapeutic efficacy of GBM is severely hindered by the blood‐brain barrier (BBB) and insufficient intratumoral penetration of therapeutics. Notably, the distance from the GBM vessel to the hypoxic regions can reach ∼230 µm, with an average of 125 µm, exceeding the penetration depth achievable by most passively diffusing drug carriers [5]. Therefore, efficient delivery of therapeutics into deep tumor regions is critical for improving GBM treatment outcomes. However, simultaneously achieving tumor targeting and deep penetration remains highly challenging.

For optimal therapeutic efficacy, therapeutics must accumulate throughout the entire tumor at a lethal dose; otherwise, surviving tumor cells (especially cancer stem cells) can repopulate the tumor when nourishment is available [6]. To reach tumor cells, therapeutic agents must flow to different regions of the tumors via a blood vessel, leave the vessel wall, penetrate through the extracellular matrix (the interstitium), and finally migrate to the target cells [7]. Unlike normal tissue, tumor vessels are highly tortuous and heterogeneously distributed, impeding the delivery of therapeutic agents to tumor cells. In addition, because tumor vessels are highly permeable and lack functional lymphatic networks, interstitial fluid pressure (IFP) increases within the tumor and decreases at its margins, creating a pressure gradient that markedly impairs convection of therapeutic agents into the tumor. Another major barrier to therapeutic penetration is the dense extracellular matrix (ECM) in the tumor microenvironment (TME), which blocks or selectively filters therapeutic agents, depending on their size, charge, morphology, and the physicochemical properties of the ECM [8], resulting in limited efficacy in the deep tumor areas and thus increasing therapeutic resistance and cancer recurrence. Consequently, nanocarrier therapeutic efficacy depends on penetration depth and intratumoral accumulation.

In passive diffusion‐based delivery systems, penetration depth is closely associated with nanocarrier size. Rational design of therapeutic nanocarriers can improve tumor penetration; however, most nanocarriers still rely primarily on passive diffusion and typically penetrate less than 100 µm from the tumor vessel, with some exceptions for ultrasmall nanoparticles [9, 10, 11]. This penetration depth remains insufficient to access hypoxic regions located 60–230 µm away from tumor vessels, depending on tumor type [5, 12].

Recently, self‐propelled micromotors/nanomotors have emerged as drug carriers that target tumors and enhance penetration depth [13, 14, 15]. Among motor types, gas‐driven motors provide sufficient propulsion and faster motion than other motors [13]. Nitric oxide (NO)‐ and oxygen (O_2_)‐driven nanomotors have been extensively investigated for deep tumor penetration by exploiting biochemical features of the GBM tumor microenvironment. Particularly, the overexpression of inducible nitric oxide synthase (iNOS) in GBM has enabled the use of arginine derivatives [16, 17] as endogenous NO‐generating substrates, while the elevated H_2_O_2_ levels in GBM have been utilized by catalase [18] or nanocatalysts [19] to generate O_2_ for propulsion. Although NO‐driven nanomotors provide an attractive strategy for tumor‐targeted propulsion, their performance remains limited by the spatial heterogeneity of iNOS expression, the rapid diffusion and clearance, and the short lifetime of NO [20, 21]. In addition, current gas‐driven motors have a relatively large size which is unfavorable for accumulation in tumors and for facilitating BBB crossing; nanomotors are typically modified with ligands that specifically target brain endothelial cells [17] or camouflaged with cell membranes [18, 19, 22, 23], which requires sophisticated chemical conjugations or coating procedures.

To address these challenges, we designed and synthesized an ultrasmall, oxygen‐propelled nanomotor composed of human heavy chain ferritin (HFn) and nanoceria (CeO_2_) (Scheme 1a). Studies have demonstrated that human heavy chain ferritin nanocage (HFn) possess BBB‐traversing and GBM‐targeting capabilities. By exploiting the overexpression of transferrin receptor‐1 (TfR‐1) on both endothelial cells and GBM cells, HFn enables efficient BBB translocation and targeted delivery to GBM tissues [24, 25]. The CeO_2_ component catalyzes the decomposition of H_2_O_2_ in the TME, generating O_2_ bubbles on the CeO_2_‐facing side, producing asymmetric propulsion and directional movement toward the H_2_O_2_ gradient (Scheme 1b). In 3D spheroid penetration and in vitro antitumor assays (loaded with doxorubicin), the nanomotor exhibited superior tumor penetration and enhanced therapeutic efficacy in the GBM spheroids.

**SCHEME 1:**
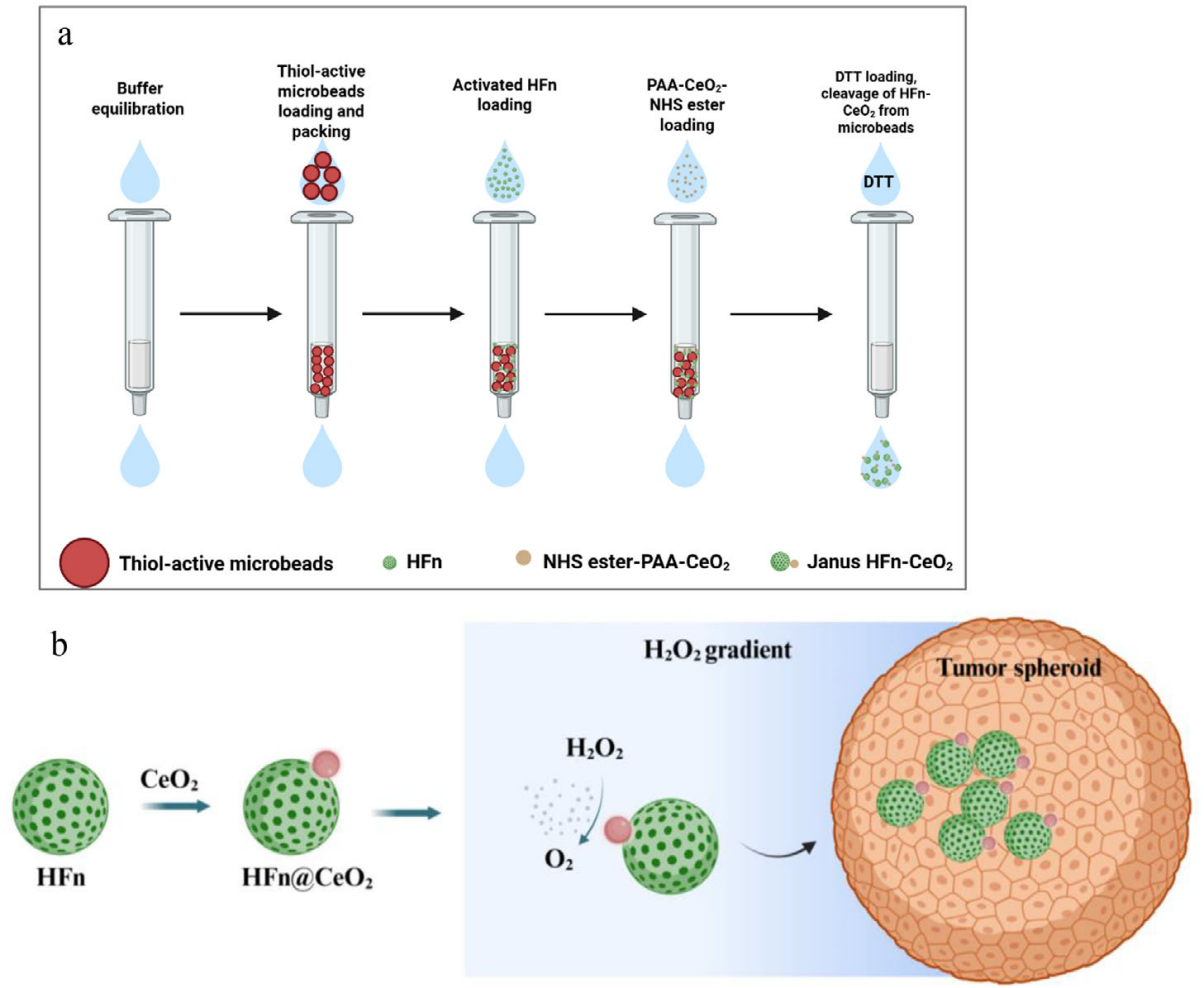
Illustration of the fabrication process of HFn@CeO_2_ nanomotor (a) and oxygen‐propelled deep penetration (b). The Scheme was prepared with Biorender.

## 2 Results and Discussion

### 2.1 Nanomotor Synthesis and Characterizations

As shown in Figure 1a, the transmission electron microscopy (TEM) image revealed that HFn has a well‐defined spherical morphology with a diameter of ∼ 10 nm while ultrasmall CeO_2_ has a diameter of ∼ 2 nm (Figure 1b). TEM imaging of HFn@CeO_2_ showed electron‐dense domains associated with the ferritin nanoparticles, consistent with the surface conjugation of CeO_2_ (Figure 1c). Atomic force microscopy (AFM) analysis provided complementary morphological characterization of HFn@CeO_2_, showing nanoparticles with a size of ∼ 18 nm (Figure 1d). The UV–vis absorbance spectra displayed characteristic peaks at 280 nm for HFn and ∼ 310 nm for CeO_2_, confirming the successful synthesis of the nanomotor (Figure S1a). The catalase activity of the synthesized nanomotor was examined using the Amplex Red Hydrogen Peroxide Assay Kit and visual observation. The results showed that H_2_O_2_ consumption increased with nanomotor concentration. Additionally, visible oxygen bubble formation in the microcentrifuge tube further demonstrated the catalytic decomposition of H_2_O_2_ (Figure S1b).

**FIGURE 1:**
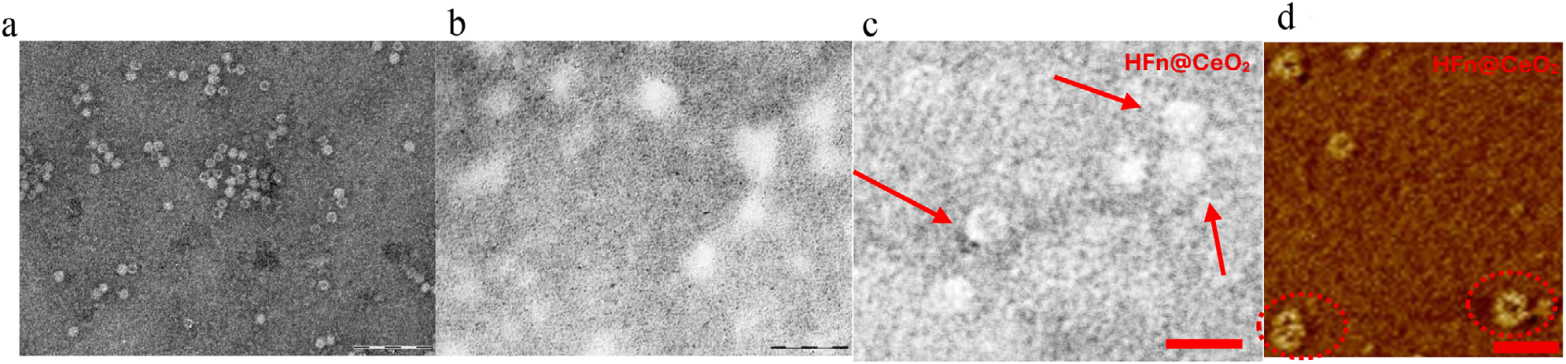
Characterization of synthesized nanoparticles. (a) TEM image of HFn. (b) TEM image of synthesized ultrasmall CeO_2_. (c,d) TEM and AFM images of the synthesized HFn@CeO_2_ nanomotor. Scale bar: 20 nm.

### 2.2 Cellular Uptake Assay

A previous study revealed that the cellular distribution of HFn NPs is cell‐type‐dependent. HFn nanoparticles undergo transcytosis in human BBB ECs, while they tend to accumulate in the lysosomes of U87 cells [24]. To investigate the cellular distribution of HFn@CeO_2_ NPs, an internalization study was performed in human brain microvascular endothelial cells (HBMECs) and U87 cells. After 4 h of incubation, green fluorescence‐labeled nanomotors were observed in the cytoplasm and nuclear region of HBMECs, while in U87 cells, nanomotors predominantly accumulated in lysosomes, as indicated by their colocalization with red fluorescence‐labeled lysosomes, with a higher Pearson's coefficient (0.85) (Figure 2). These results suggest that HFn@CeO_2_ exhibits a cellular internalization pattern similar to that observed in HFn, which may preserve its BBB‐traversing capacity.

**FIGURE 2:**
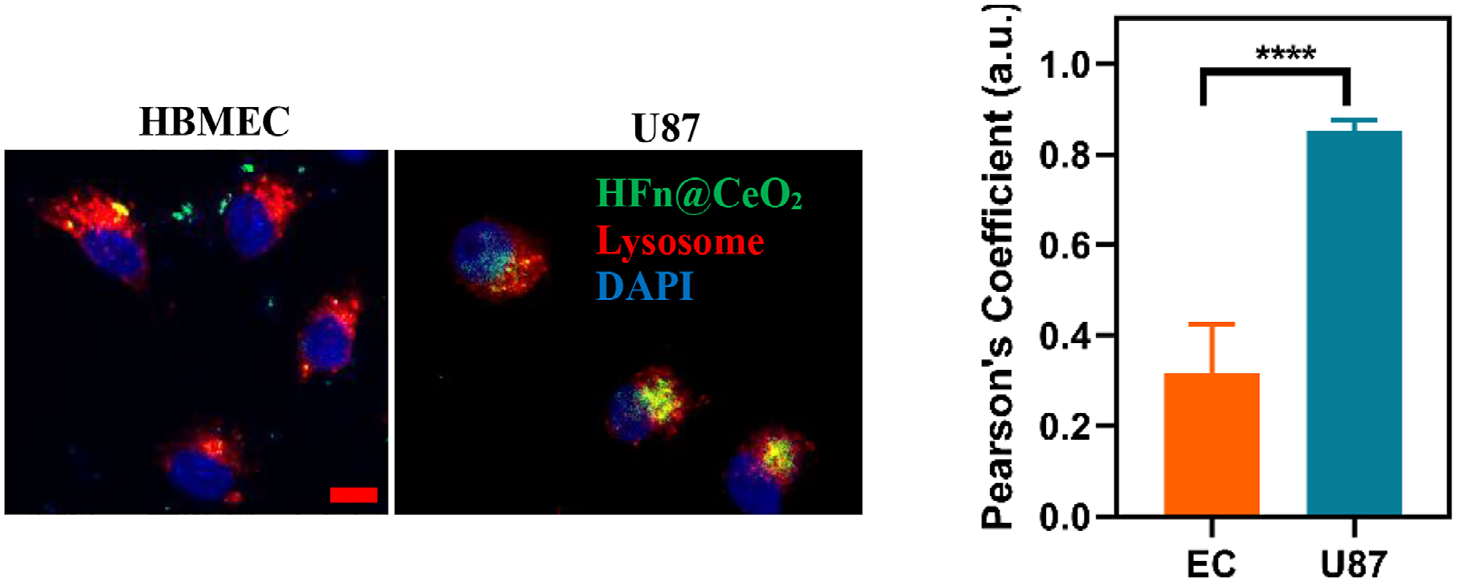
Colocalization of HFn@CeO_2_ and lysosomes in ECs and U87 tumor cells. Nanomotors were labeled with Alexa Fluor 488, and lysosomes were stained with LysoBrite Red DND‐99. Cell nuclei were stained with DAPI. Statistical significance was analyzed with Student‐t test (*n* = 10). ^****^ *p* < 0.0001. Scale bar: 20 µm.

### 2.3 In Vitro Transwell BBB Assay and Spheroid Accumulation

Both HBMECs of BBB and GBM cells overexpress Trf‐1 receptors. HFn nanocages can cross the BBB through Trf‐1 receptor‐mediated transcytosis. To determine whether HFn@CeO_2_ nanomotors affect the BBB‐traversing capacity of HFn, an in vitro transwell BBB model was employed. As illustrated in Figure 3a, HBMECs were seeded on the top of the inserts, and after the cells became confluent, the nanomotor solution was added. The immunofluorescent staining of tight junction protein zonula occludens‐1 (ZO‐1) showed that an intact endothelial monolayer was formed on the insert (Figure 3b).

**FIGURE 3:**
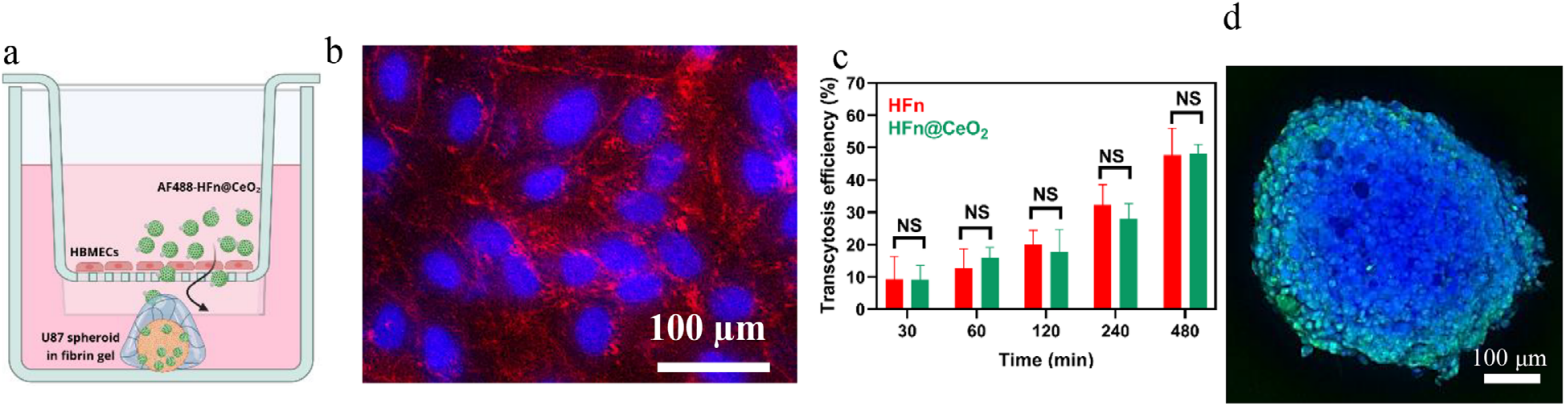
In vitro transwell BBB assay: (a) Illustration of in vitro transwell BBB model; (b) immunofluorescent staining of ZO‐1 protein of HBMECs on the insert; (c) The BBB‐crossing efficiency over time (*n* = 3); (d) Accumulation of HFn@CeO_2_ in spheroid after crossing the transwell membrane. Statistical significance was analyzed with the Student t‐test. Scale bar: 100 µm. NS: no significance.

After the addition of Alexa Fluor 488 (AF488)‐labeled HFn and HFn@CeO_2_, both nanocarriers effectively traversed the BBB. After 2 h of incubation, 17.7% ± 6.8% of HFn@CeO_2_, crossed the barrier, increasing to 48.1% ± 3.0% by 8 h. Statistical analysis indicated that the conjugation of CeO_2_ to HFn did not significantly alter its BBB‐crossing capability (Figure 3c). To further visualize the nanomotors after traversal of the endothelial barrier, U87 spheroids were placed in the lower compartment of the transwell system. Following overnight incubation, fluorescence from HFn@CeO2 was observed within the U87 spheroids, indicating that the nanomotors had traversed the endothelial monolayer and subsequently accumulated in the tumor spheroids (Figure 3d).

After traversal across the BBB, the biosafety of CeO_2_ in the brain microenvironment is an important consideration. Previous studies have demonstrated that the antioxidant and neuroprotective properties of cerium oxide nanoparticles in neurological applications including long‐duration space missions and related neurodegenerative conditions [26, 27]. More importantly, little evidence of toxicity has been reported at doses relevant to the treatment of neurodegenerative diseases, supporting the potential neurobiocompatibility of CeO_2_‐based nanomaterials [28].

### 2.4 Chemotactic Behavior of HFn@CeO_2_ Nanomotor

To evaluate directional movement of the synthesized nanomotors, a chemotaxis assay was performed in a petri dish. As illustrated in Figure 4a, a pre‐soaked agarose cylinder serves as a localized H_2_O_2_ source and is placed at the edge of the Petri dish, whereas nanomotors are added to the center. Samples were collected from pre‐determined locations, and the nanomotor distribution was analyzed based on fluorescence intensity. At time 0, the nanomotors were evenly distributed around the center (Figure 4b). After 15 min, a clear migration toward the H_2_O_2_ source was observed, indicating directional movement along the H_2_O_2_ gradient (Figure 4c). This result demonstrates that the synthesized nanomotor exhibits chemotactic behavior toward H_2_O_2_. The fluorescence distribution under an H_2_O_2_ gradient showed directional migration of HFn@CeO_2_. Given the small size of the nanomotors, direct tracking of individual particles is technically challenging, and the fluorescence‐based assay was used to evaluate their overall migration behavior rather than individual particle trajectories.

**FIGURE 4:**
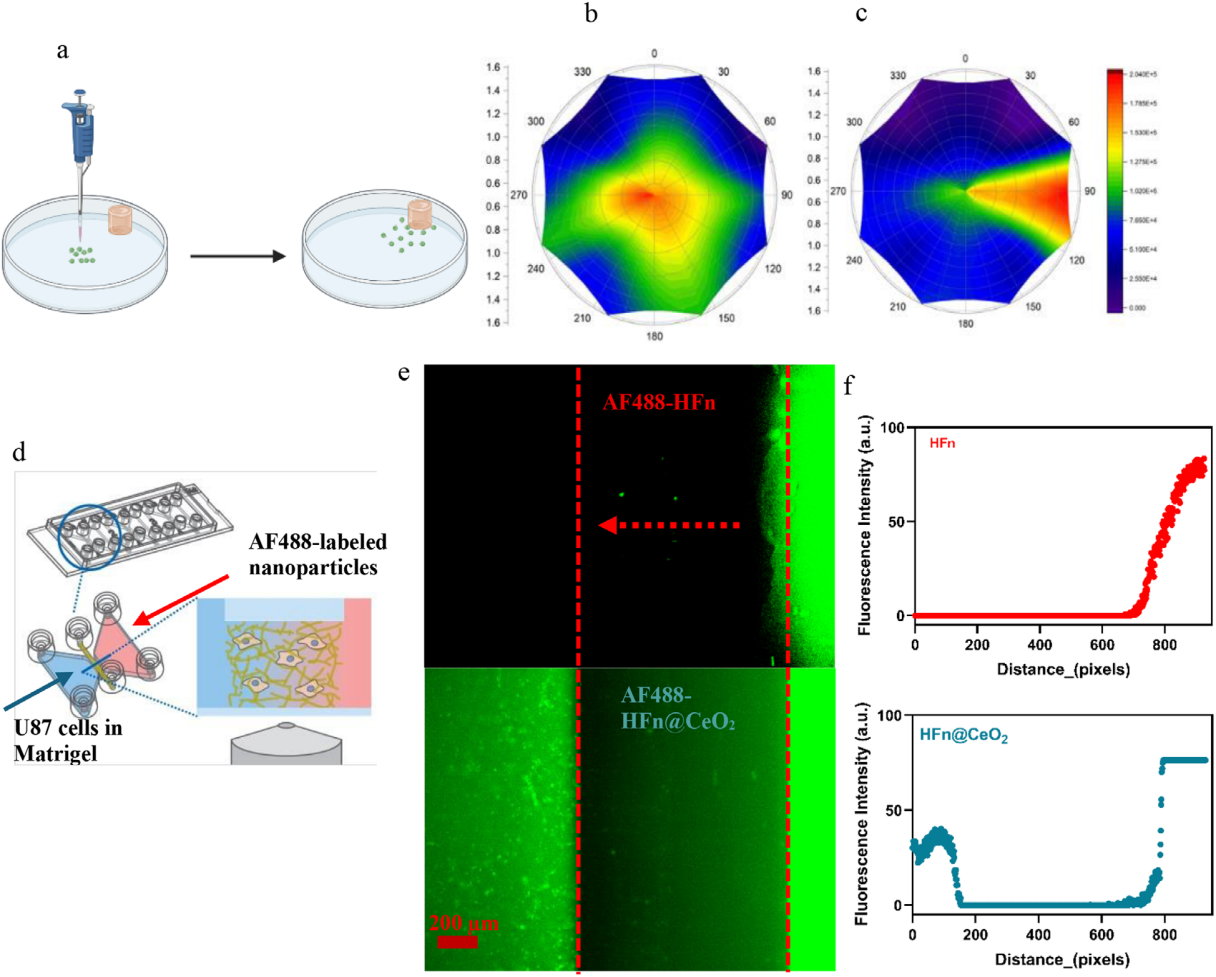
Chemotactic behavior of HFn@CeO_2_ nanomotor: (a) Illustration for experimental settings; H_2_O_2_‐soaked agarose cylinder was located at the right edge of the petri dish; The distribution of HFn@CeO_2_ at 0 min (b) and 15 min (c) after HFn@CeO_2_ nanomotors were added at the center of the petri dish. (d) Experimental settings for chemotaxis assay in Matrigel‐embedded cells. (e) AF488‐labeled nanoparticles in the right chamber migrated toward the U87 cells in the left chamber. Scale bar: 200 µm. (f) Line profiles of average fluorescence intensity across the middle chamber.

Next, we also assessed whether the nanomotors migrate toward glioblastoma cells, given the elevated H_2_O_2_ levels in the tumor microenvironment. A µ‐slide chemotaxis device is used to track the movement of the nanomotors (Figure 4d). U87 cells mixed with an equal volume of Matrigel and seeded into the left chamber, and AF488‐labelled nanoparticle solution (1 µg/mL AF488 concentration) was added to the right chamber. After overnight incubation, nanomotors in the right chamber migrated toward the U87 cells in the left chamber (Figure 4e) while HFn had limited diffusion across the middle chamber, suggesting the chemotactic behavior of HFn@CeO_2_ toward tumor cells. The line graphs of fluorescence intensity across the middle chamber also confirmed the migration of nanomotors (Figure 4f).

### 2.5 Penetration and Distribution of Nanomotors in Spheroid

Spheroids with a large diameter (> 400 µm) display a layered structure consisting of a necrotic core surrounded by a viable layer of quiet cells and an outer rim of proliferating cells, creating physiological gradients, such as oxygen, nutrient, and pH gradients [29]. Central cells adapt their metabolism in response to microenvironmental stresses generated by diffusive gradients and acquire clinically relevant resistance to chemotherapy‐induced apoptosis [30, 31, 32, 33]. Therefore, deep delivery into solid tumor tissue is critical for therapeutic efficacy.

After we demonstrated the chemotactic behavior of HFn@CeO_2_ nanomotor toward H_2_O_2_, a penetration assay was conducted in large spheroids. U87 spheroids were scanned from bottom to top, and HFn nanocarriers showed limited distribution in the central slices, with a penetration depth of 91.7 ± 17.1 µm, and accumulated in the peripheral region, consistent with a previous study [34]. In contrast, HFn@CeO_2_ nanomotors were well distributed throughout the spheroid core (Figure S2 and Figure 5a). Line profiles further confirmed the deeper penetration of HFn@CeO_2_ nanomotors, with the more fluorescence concentrated toward the center of spheroids (Figure 5b).

**FIGURE 5:**
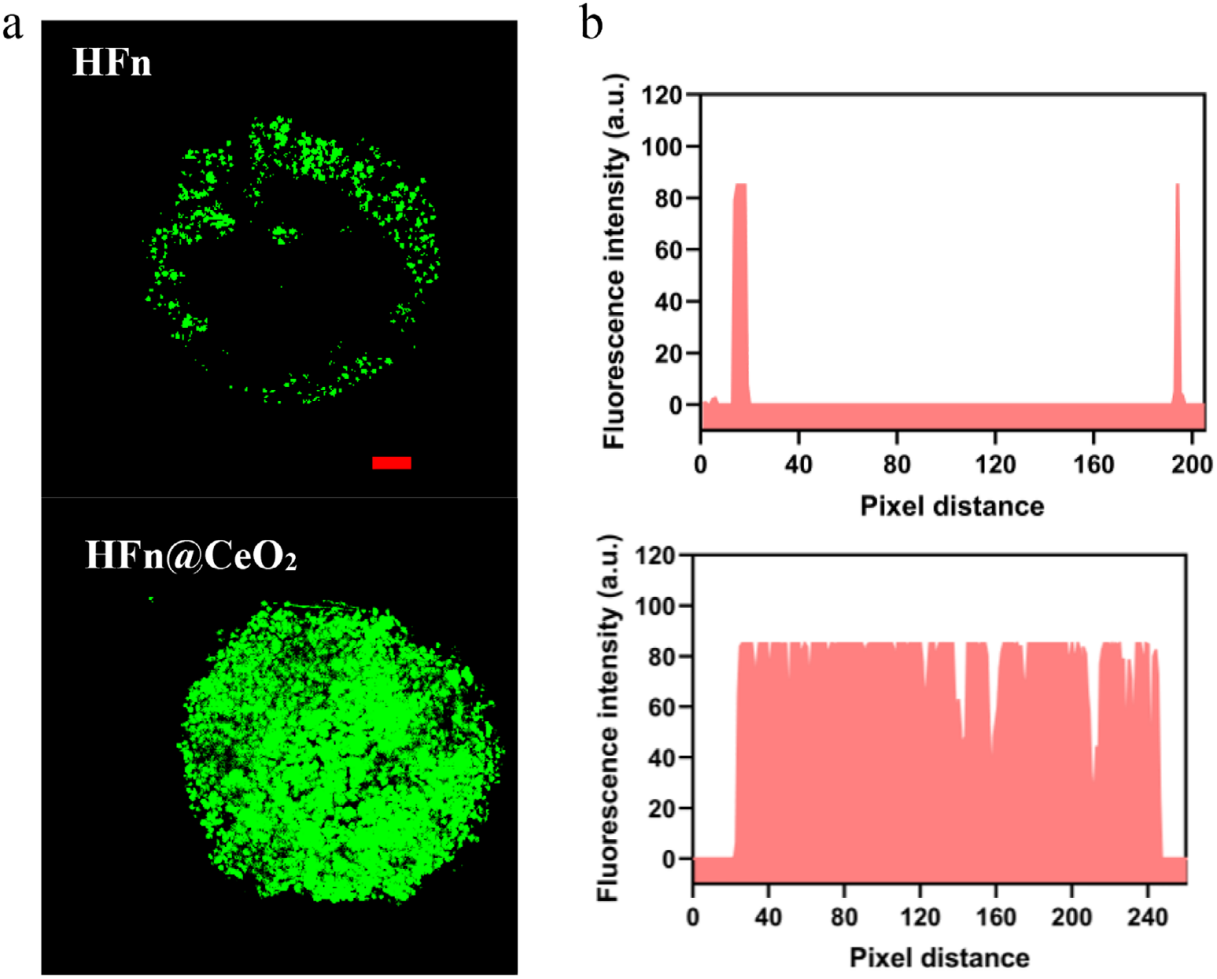
Penetration of nanoparticles in U87 spheroids. (a) Representative images of central cross section of spheroids after incubating with nanoparticles. (b) Line profiles of central cross section of spheroids incubated with nanoparticles (*n* = 4). Scale bar: 100 µm.

### 2.6 Formulation of DOX‐Loaded Nanocarriers

Next, we evaluated the feasibility of HFn and HFn@CeO_2_ as drug carriers. UV–vis spectra and fluorescence spectra were used to characterize doxorubicin (DOX)‐loaded nanocarriers. After loading with DOX, nanocarriers had a characteristic absorption peak at ∼ 482 nm (Figure S3a) and a characteristic fluorescence peak at ∼ 592 nm (Figure S3b). The loading efficiency and loading capacity were calculated as follows:

$$\begin{array}{ccc} & & Loadingefficiency\left(\%\right)\\ & & =\left(weight\;of\;loaded\;DOX\right)/\left(weight\;of\;added\;DOX\right)x100 \end{array}$$

$$\begin{array}{ccc} & & Loading\;capacity\;\left(\%\right)=\left(weight\;of\;loaded\;DOX\right)/\\ & & \;\left(total\;weight\;of\;loaded\;DOX+drugcarrier\right)x100 \end{array}$$

For HFn, the loading efficiency was 38.5%, and the loading capacity was 10.4%. After the conjugation process, DOX content decreased in HFn@CeO_2_‐DOX, with a loading efficiency of 19.0% and a loading capacity of 5.4%. The reduced DOX loading after CeO_2_ conjugation may be associated with partial drug loss during the subsequent conjugation and purification steps, as DOX was loaded into HFn prior to CeO_2_ conjugation.

After confirming the distribution of HFn@CeO_2_ nanomotors within tumor spheroids, the intratumoral distribution of DOX released from HFn‐DOX and HFn@CeO_2_‐DOX was also assessed. Spheroids were treated with DOX, HFn‐DOX, and HFn@CeO_2_‐DOX (equivalent DOX concentration: 1 µg/mL) overnight and analyzed using confocal microscopy. As shown in Figure 6, both free DOX and HFn‐DOX exhibited limited penetration, primarily accumulating at the spheroid periphery, with penetration depth of 19.3 ± 6.8 µm and 48.8 ± 15.1 µm, respectively. In contrast, HFn@CeO_2_‐DOX demonstrated significantly deeper distribution and accumulation in the spheroid core, with a penetration depth of 125.4 ± 11.2 µm.

**FIGURE 6:**
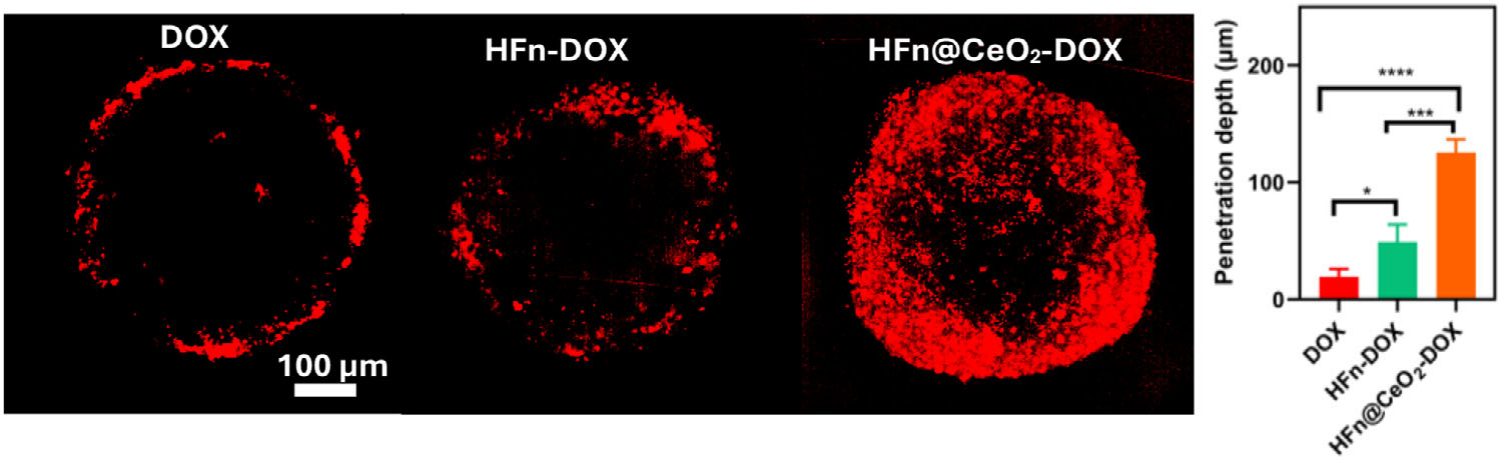
The distribution of delivered DOX in spheroids. Representative reconstructed images of free DOX (left), HFn‐DOX (middle), and HFn@CeO_2_‐DOX (right). DOX has intrinsic fluorescence, and the cell nuclei were stained with DAPI. Statistical significance was analyzed with One‐way ANOVA using GraphPad Prism 9 (*n* = 4). ^*^ *p* < 0.05, ^***^ *p* < 0.001, ^****^ *p* < 0.0001.

### 2.7 In Vitro Anti‐Tumor Assay

Next, the antitumor effects of delivered DOX were evaluated in green fluorescence protein (GFP)‐expressing U87 (GFP‐U87) spheroids. As shown in Figure S4, empty HFn and HFn@CeO_2_ nanocarriers exhibited no cytotoxicity after 7 days of treatment. In contrast, HFn@CeO_2_‐DOX exhibited notably enhanced antitumor activity, with IC_50_ values of 1.75 µM for free DOX, 1.13 µM for HFn‐DOX, and 0.33 µM for HFn@CeO_2_‐DOX (Figure 7). An extra sum‐of‐squares F‐test showed a significant overall difference in the fitted IC_50_ values among the three treatment groups (F(2,64) = 5.905, *p* = 0.0044). The superior antitumor efficacy of HFn@CeO_2_‐DOX is likely due to the improved tumor penetration.

**FIGURE 7:**
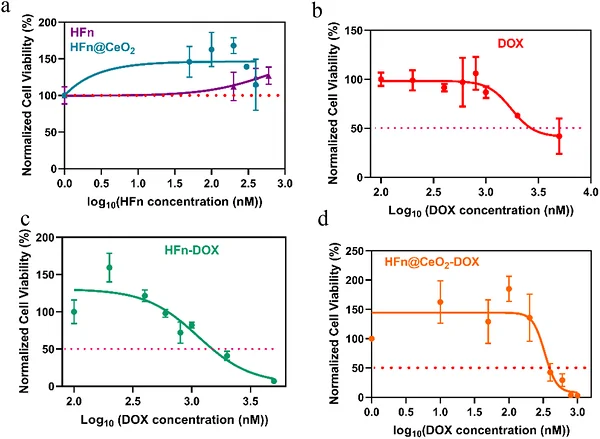
In vitro antitumor assay of various nanocarriers in spheroids. Cells were treated with empty nanocarriers, free DOX, and DOX‐formulated nanocarriers, and cell viability was normalized with the GFP fluorescence intensity of control groups without any treatment. IC_50_ values were analyzed with GraphPad Prism 9 (*n* = 4).

## 3 Conclusions

In summary, an ultrasmall nanomotor comprising the targeting protein HFn and the catalyst CeO_2_ was successfully synthesized based on a computational design framework for nanoparticle transport in GBM tissue. The nanomotors exhibited chemotactic behavior and actively migrated toward H_2_O_2_ and tumor cells in dedicated in vitro chemotaxis assays. In a transwell BBB model, the nanomotor effectively crossed the barrier, preserving the BBB‑traversing capability of HFn while introducing propulsion via H_2_O_2_. The enhanced in vitro antitumor efficacy is evidenced by a low IC_50_ value, 3.4 times lower than HFn‐DOX and 4.3 times lower than free DOX, likely attributed to its deep penetration capability. Overall, these findings establish a chemotactic nanomotor platform that integrates modeling‑guided design, BBB‑mimicking transport, and deep penetration in GBM spheroids, providing in vitro proof‑of‑concept and motivating future in vivo validation in brain tumor models.

## 4 Experiments

### 4.1 Materials

Cerium (III) nitrate hexahydrate (Ce(NO_3_)_3_.6H_2_O), polyacrylic acid (PAA, average Mw ∼2,000), 28%–30% ammonium hydroxide solution, and Amicon Ultracentrifuge unit (30 kDa molecular weight cutoff) were purchased from Sigma–Aldrich. High‐capacity thiol reactive resin was obtained from Nanocs. Alexa Fluor 488‐NHS ester, Zebra spin desalting columns (40 K molecular weight cutoff), and 10 mL Pierce centrifuge columns were purchased from Thermo Fisher Scientific. Human heavy chain ferritin (HFn) was a kind gift from Dr. Fadi Bou‐Abdallah at SUNY Potsdam.

### 4.2 Nanoceria and HFn@CeO_2_ Synthesis

PAA‐CeO_2_ synthesis: PAA‐modified CeO_2_ was prepared as described in a previous report [35]. Briefly, 1 M Ce(NO_3_)_3_.6H_2_O solution was mixed with 0.5 M PAA solution. Under stirring, the mixture was added to 30 mL ammonium hydroxide solution. After stirring for 24 h, nanoparticles were centrifuged at 4,000 rpm for two 30 min cycles to remove large agglomerates. The supernatant was purified using an Amicon cell (30K, Sigma–Aldrich).

Conjugation of HFn to agarose beads: Thiol‐active 6B beads (diluted 100 times with deionized water) were hydrated with DI water in 10 mL Pierce centrifuge column and then equilibrated with 4–5 column volumes of reaction buffer (5 mM NaHPO_4_, 100 mM NaCl, 5 mM EDTA, pH 7.0). 300 µg of HFn in 300 µL PBS was activated with TCEP (> 100 times molar ratio, TCI Chemicals) at room temperature for 2 h. Then desalting columns were used to remove excess TCEP and purified HFn was added to the slurry of beads. The mixture was incubated at 4°C overnight. Unattached HFn was washed away with 4–5 column volumes of reaction buffer [36].

Conjugation of PAA‐CeO_2_ to HFn: 100 µL PAA‐CeO_2_ was treated with 30 mM EDC (Thermo Fisher Scientific) and 6 mM NHS (Thermo Fisher Scientific) in ethanol. After 2 h of incubation at room temperature, excess EDC/NHS was removed by centrifugation. The activated PAA‐CeO_2_ was dispersed in PBS, added to HFn‐conjugated bead slurry, and incubated at room temperature for 4 h.

Release of HFn@CeO_2_ from the bead slurry: 50 mM dithiothreitol (DTT, Thermo Fisher Scientific) was added to the slurry mixture and incubated at room temperature for 1 h. Then released HFn@CeO_2_ was collected, and the centrifuge column was eluted with PBS for 3–4 times. After collection, HFn@CeO_2_ nanomotors were purified using Zebra spin desalting columns (Thermo Fisher Scientific). The concentration of protein in of HFn@CeO_2_ nanomotor was quantified using Bradford protein assay based on prepared calibration curve.

### 4.3 Instrumentation and Characterizations

The morphology of HFn and synthesized PAA‐CeO_2_ were characterized by transmission electron microscopy (FEI Morgagni 260). The morphology of nanomotor was characterized by atomic force microscopy (AFM, Bruker MultiMode 8‐HR). The absorbance and fluorescence were measured using a microplate reader (Tecan Infinite 200 Pro M Plex).

### 4.4 Fluorescence Labeling of HFn and HFn@CeO_2_

The labeling of HFn and HFn@CeO_2_ with Alexa Fluor 488 (AF488, Thermo Fisher Scientific) was followed a previous protocol [37]. 100 nM Alexa Fluor 488 NHS ester dye was added to 50 nM HFn or HFn@CeO_2_ solution in 1 mL PBS. The mixture was incubated at room temperature for 4 h. After purification of nanoparticle using desalting columns, samples were collected and stored at 4°C under dark for further use.

### 4.5 Drug Loading into Nanomotor

Loading of doxorubicin (DOX, Sigma–Aldrich) into HFn was completed by one‐step encapsulation according to a previous study [38]. 1 mg HFn or HFn@CeO_2_ (equivalent of HFn content) in 1 mL of 50 mM Tris‐HCl buffer (pH 9.0, Thermo Fisher Scientific) was mixed with 0.3 mg DOX and incubated at 60°C water bath for 1 h. After purified with desalting columns, DOX‐formulated nanocarriers were stored at 4°C under dark for further studies.

### 4.6 Cell Culture

Human glioblastoma cell line U87‐MG (ATCC) and GFP‐expressing U87 (GFP‐U87, Angio‐Proteomie) were cultured in Eagle's modified essential medium (EMEM, ATCC), supplemented with 10% fetal bovine serum (FBS, Thermo Fisher Scientific) and 1% penicillin‐streptomycin. Human brain microvascular endothelial cells (HBMECs, ScienCell) endothelial cell medium kit. Cells were maintained in 5% CO_2_ incubator at 37°C with 95% relative humidity until ∼ 80% confluence. Cells were detached with 0.05% Trypsin‐EDTA solution and neutralized with cell culture medium. Subsequently, cells were collected for further assays.

### 4.7 Cellular Uptake

HBMECs and U87 cells were seeded onto poly‐L‐lysine‐coated coverslips in a 24‐well plate and incubated for 12 h to allow attachment. After 1 µM AF488‐labeled HFn and HFn@CeO_2_ were added and incubated for 4 h, cells were stained with LysoBrite Red DND‐99 (AAT Bioquest) for 30 min at 37°C, washed with PBS, and fixed in 4% cold paraformaldehyde (PFA, Thermo Fisher Scientific) solution for 30 min. Cell nuclei were stained with 1 µg/mL 4’,6‐diamidino‐2‐phenylindole (DAPI, Thermo Fisher Scientific) for 30 min at room temperature. Z‐stack scanning of the fluorescent images were captured with BioTek Lionheart LX fluorescent microscopy. Colocalization of nanoparticles and lysosomes were analyzed using JACoP plugin in ImageJ (NIH).

### 4.8 In Vitro BBB Penetration and Spheroid Accumulation

400,000 HBMECs were seeded into the inserts of Corning Transwell 6‐well plates with a surface are of 4.67 cm^2^ and pore size of 8 µm. After 3‐day incubation, cells were fixed with 4% PFA, stained with Dil dye (AAT Bioquest), and stained with 10 µg/mL DAPI. Fluorescent images were acquired using Lionheart automated microscopy.

For the nanoparticle traversing assay, AF488‐labeled HFn and HFn@CeO_2_ were added to the upper inserts. At 30 min, 60 min, 120 min, 240 min, and 480 min, solutions at the bottom were collected, and fluorescence intensity was analyzed using a microplate reader (Tecan Infinite 200 Pro M Plex). The penetration efficacy was calculated based on AF488 calibration.

For the spheroid accumulation assay, U87 spheroids were embedded in fibrin gel and placed at the bottom of the transwell plate. After overnight incubation, the spheroids were fixed with 4% PFA and stained with DPAI. The accumulation of AF488‐labelled nanoparticles in spheroids was analyzed using confocal microscopy (ImageXpress, Molecular Devices).

### 4.9 Chemotaxis Assay

An agarose cylinder gel (5 mm in diameter, 1 cm in height) was soaked in 10 mM H_2_O_2_ solution (Thermo Fisher Scientific) for 2 h at room temperature. The H_2_O_2_‐loaded agarose cylinder was then placed at the edge of a 35 mm Petri dish (Thermo Fisher Scientific) containing 2 mL of PBS. Immediately afterward, 10 µL of AF488‐labeled HFn@CeO_2_ solution (protein concentration: 150 µg/mL) was added to the center of the dish. 10 µL samples were collected at predetermined locations at time points 0 and 15 min. The fluorescence intensity of each sample was measured using a microplate reader (Tecan Infinite 200 M Plex) and a heatmap was generated to visualize the spatial distribution of the nanomotors.

Another chemotaxis assay was conducted in µ‐slide chemotaxis chamber (ibidiTreat, ibidi). U87 cells at the density of 1 × 10^6^ cells/mL were mixed with equal volume of Matrigel solution (Corning) and injected into the left chamber. After incubation at 37°C for 30 min to solidify the Matrigel, fresh EMEM medium was injected into the left chamber. After overnight incubation, EMEM medium was injected into the middle channel and AF488‐labeled HFn@CeO_2_ nanomotor solution (1 µg/mL AF488) were injected into the right chamber. After 12 h incubation, the migrated nanomotors were imaged using fluorescence microscopy (Biotek, Lionheart).

### 4.10 Nanomotor Penetration in Spheroids

U87 cells were seeded into ultra‐low attachment 96‐well plate (PrimeSurface, S‐bio) at 10,000 cells per well and incubated for 3 days. Spheroids were treated with AF488‐labeled HFn and HFn@CeO_2_ (equivalent of fluorescence intensity) overnight. Afterward, spheroids were fixed with 4% PFA and stained with 10 µg/mL DAPI. Fluorescent images were captured using confocal microscopy (ImageXpress, Molecular Devices).

For DOX distribution, free DOX, HFn‐DOX, and HFn@CeO_2_‐DOX (1 µg/mL DOX) were added to spheroids and incubated overnight. Spheroids were then fixed by 4% PFA and stained with 10 µg/mL DAPI solution. Fluorescent images were acquired with confocal microscopy (ImageXpress, Molecular Devices).

### 4.11 Antitumor Assay in Spheroids

GFP‐U87 cells were seeded into ultra‐low attachment 96‐well plate (PrimeSurface, S‐bio) at 10,000 cells per well and incubated for 3 days to form spheroids. Spheroids were then treated with DOX, HFn‐DOX, and HFn@CeO_2_‐DOX (at equivalent of DOX concentrations) for 7 days. Similarly, spheroids were also treated with empty nanocarriers (HFn and HFn@CeO_2_) as control groups. After treatment, spheroids were fixed with 4% PFA and stained with 10 ug/mL DAPI. Fluorescent images were captured using fluorescence microscopy and fluorescence intensity was quantified using Image J (NIH). Cell viability was determined based on GFP fluorescence intensity and normalized to the untreated control group.

### 4.12 Statistical Analysis

All data were presented as Mean ± Standard Deviation, and statistical comparisons were performed using Student t‐test and One‐way ANOVA with an alpha value of 0.05. *p* value less than 0.05 was considered as statistically significant. Dose‐response curves were fitted using a four‐parameter variable‐slope nonlinear regression model. Differences in fitted IC_50_ values among treatment groups were evaluated using an extra sum‐of‐squares F‐test. All data were analyzed using GraphPad Prism 9 and Origin 2021.

## Author Contributions

**Jun Ma**: Conceptualization, investigation, writing. **Ruochen Liu**: Investigation (AFM measurement). **Ibrahim Chamseddine**: investigation. **Jingjing Qiu**: Conceptualization, supervision, review. **Shiren Wang**: Conceptualization, writing, review, supervision, funding acquisition.

## Conflicts of Interest

The authors declare no conflicts of interest.

## Supporting information




**Supporting File**: mabi70267‐sup‐0001‐SuppMat.docx.

## Data Availability

The data that support the findings of this study are available from the corresponding author upon reasonable request.
