# Supplementary material for: Ferritin‐Based Nanomotors for Deep Glioblastoma Penetration and Enhanced Drug Delivery
Source: Macromol Biosci. 2026 Sep 30;26(10):e70267. doi: 10.1002/mabi.70267 (PMC13625947; doi:10.1002/mabi.70267)
Supplement: Supplementary file 1 [file MABI-26-e70267-s001.docx]

Figure S1. Characterization of synthesized nanoparticles. (a) UV-Vis absorbance spectra of HFn@CeO_2_ nanomotor. (b) H_2_O_2_ consumption by HFn@CeO_2_ measured by Amplex Red hydrogen peroxide assay kit and digital image of O_2_ bubbles produced by HFn@CeO_2_.

a

b

HFn

Bottom

Top

HFn@CeO_2_

Top

Bottom

Figure S2. Characterization of synthesized nanoparticles. (a) UV-Vis absorbance spectra of HFn@CeO_2_ nanomotor. (b) H_2_O_2_ consumption by HFn@CeO_2_ and digital image of O_2_ bubbles produced by HFn@CeO_2_. Scale bar: 200 µm.

a

b

Figure S3. Characterization of DOX-loaded HFn and HFn@CeO_2_. UV-Vis absorbance spectra (a) and fluorescence spectra (b) of empty nanocarriers and DOX-loaded nanocarriers.

**HFn-DOX**

**0.2 µM**

**0.4 µM**

**0.6 µM**

**0.8 µM**

**1 µM**

**2 µM**

**5 µM**

**Control** (Spheroid without any treatment)

**Control** (Spheroid without any treatment)

**DOX**

**0.2 µM**

**0.6 µM**

**0.8 µM**

**1 µM**

**2 µM**

**5 µM**

**HFn@CeO_2_**

**0.1 µM**

**0.2 µM**

**0.3 µM**

**0.4 µM**

**0.6 µM**

**Control** (Spheroid without any treatment)

**HFn**

**0.2 µM**

**0.4 µM**

**0.6 µM**

**2 µM**

**5 µM**

**0**

**Control** (Spheroid without any treatment)

**Control** (Spheroid without any treatment

**0.1 µM**

**0.2 µM**

**0.4 µM**

**0.6 µM**

**0.8 µM**

**1 µM**

**2 µM**

**5 µM**

**HFn@CeO_2_-DOX**

Figure S4. Representative images of GFP-U87 spheroids treated with various nanocarriers and DOX-formulated nanocarriers for 7 days. The concentration range of nanocarriers was from 0 to 5 µM. The control groups were spheroids without any treatment. HFn, DOX, and HFn-DOX were evaluated in the same experimental batch and therefore shared the same representative control image. Similarly, HFn@CeO_2_ and HFn@CeO_2_-DOX were evaluated in a separate experimental batch and accordingly shared the same representative control image. Scale bar: 100 µm.
